# Dendrobium alkaloids prevent Aβ_25–35_-induced neuronal and synaptic loss via promoting neurotrophic factors expression in mice

**DOI:** 10.7717/peerj.2739

**Published:** 2016-12-13

**Authors:** Jing Nie, Yong Tian, Yu Zhang, Yan-Liu Lu, Li-Sheng Li, Jing-Shan Shi

**Affiliations:** 1 Shanghai University of Traditional Chinese Medicine, Shanghai, China; 2 Department of Pharmacology and the Key Laboratory of Basic Pharmacology of Guizhou Province, Zunyi Medical College, Zunyi, Guizhou Province, China

**Keywords:** Alzheimer’s disease, Neuron, Synaptic loss, *Dendrobium nobile* Lindl. alkaloids, Neurotrophic factor, Apoptosis

## Abstract

**Background:**

Neuronal and synaptic loss is the most important risk factor for cognitive impairment. Inhibiting neuronal apoptosis and preventing synaptic loss are promising therapeutic approaches for Alzheimer’s disease (AD). In this study, we investigate the protective effects of Dendrobium alkaloids (DNLA), a Chinese medicinal herb extract, on β-amyloid peptide segment 25–35 (Aβ_25-35_)-induced neuron and synaptic loss in mice.

**Method:**

Aβ_25–35_(10 µg) was injected into the bilateral ventricles of male mice followed by an oral administration of DNLA (40 mg/kg) for 19 days. The Morris water maze was used for evaluating the ability of spatial learning and memory function of mice. The morphological changes were examined via H&E staining and Nissl staining. TUNEL staining was used to check the neuronal apoptosis. The ultrastructure changes of neurons were observed under electron microscope. Western blot was used to evaluate the protein expression levels of ciliary neurotrophic factor (CNTF), glial cell line-derived neurotrophic factor (GDNF), and brain-derived neurotrophic factor (BDNF) in the hippocampus and cortex.

**Results:**

DNLA significantly attenuated Aβ_25–35_-induced spatial learning and memory impairments in mice. DNLA prevented Aβ_25–35_-induced neuronal loss in the hippocampus and cortex, increased the number of Nissl bodies, improved the ultrastructural injury of neurons and increased the number of synapses in neurons. Furthermore, DNLA increased the protein expression of neurotrophic factors BDNF, CNTF and GDNF in the hippocampus and cortex.

**Conclusions:**

DNLA can prevent neuronal apoptosis and synaptic loss. This effect is mediated at least in part via increasing the expression of BDNF, GDNF and CNTF in the hippocampus and cortex; improving Aβ-induced spatial learning and memory impairment in mice.

## Introduction

Alzheimer’s disease (AD) is the most common type of dementia, and is characterized by progressive memory impairment and cognitive decline (Roberson & Mucke, 2006). Mounting evidence has indicated that dementia attributed to synaptic dysfunction and neuronal loss in the hippocampus and its associated cortex (Youssef et al., 2008; Selkoe, 2002; Niikura et al., 2002; Scheff et al., 2007), which are caused by the accumulation of soluble A*β* oligomers (Rowan et al., 2007; Lacor et al., 2004; Haass & Selkoe, 2007). A*β* is the major constituent in senile plaques and cerebral amyloid angiopathy, which are two most distinctive histopathologies in AD. The accumulation of A*β* in the brain initiates a cascade of events, such as activating astrocytes and microglia, initiating inflammatory responses, which lead to oxidative injury, altering neuronal ionic homeostasis, kinases/phosphatase activities, and so on (Klafki et al., 2006). These cascades result in a wide range of neuronal/synaptic dysfunction and loss, as well as loss of neurotrophin retrograde transport, therefore causing patients to present with the symptoms of dementia. Adjustment of the pathological progress of amyloid peptide is a key strategy to slow down the AD progression. In addition to reduction of the A*β* levels in the brain (Klafki et al., 2006), the concomitant application of neuroprotective agents may be the alternative therapeutics for AD, and a strategy to prevent progressive synaptic and cognitive degeneration (Klafki et al., 2006). Neurotrophins (NT) are synthesized and secreted by the target tissue, and after binding to its receptors and transported in a retrograde manner to the cell body. They exert a wide range of actions, including neuronal survival and differentiation, modulation of neuronal excitability, development and maintenance of synapses and modification of synaptic structure and function (Poo, 2001). The application of neurotrophic factors such as BDNF and CNTF could enable the modulation of neuronal survival and synaptic connectivity (Lu, Christian & Lu, 2008; Garcia et al., 2010)

*Dendrobium nobile* is a traditional Chinese herbal medicine. In our previous studies, alkaloids extract from *Dendrobium nobile* Lindl. (DNLA) showed neuro-protective activity. For example, DNLA can prevent neuronal damages induced by LPS (Li et al., 2011; Zhang et al., 2011), and oxygen-glucose deprivation and reperfusion (Wang et al., 2010), decrease neuronal apoptosis, hyperphosphorylation of tau protein (Yang et al., 2014) and A*β* deposition in rat brain (Chen et al., 2008). The present study aimed to explore the effects of DNLA in protecting neurons from Aβ_25–35_-induced neurotoxicity in mice, and analyzed the mechanism from the aspects of promoting the secretion of neurotrophic factors.

## Materials and Methods

### Drugs and reagents

**Figure 1:**
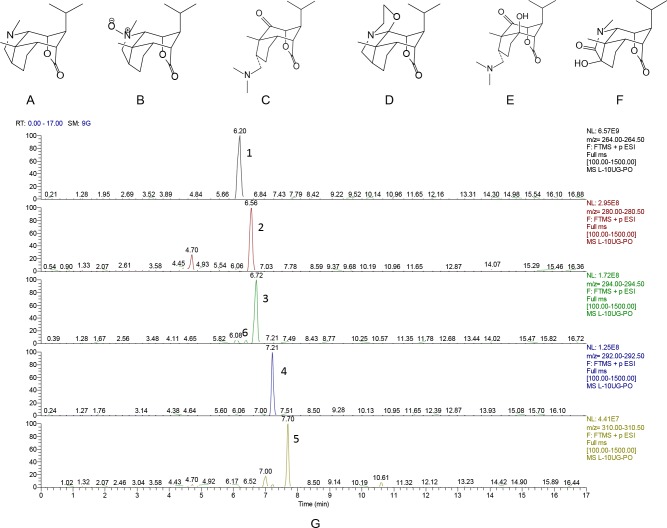
Chemical structures and chromatograms of Dendrobium alkaloids. Chemical structures and chromatograms of Dendrobium alkaloids (A) Dendrobine, (B) Dendrobine-N-oxide, (C) Nobilonine, (D) Dendroxine, (E) 6-Hydroxy-nobilonine, and (F) 13-Hydroxy-14-oxodendrobine. (G) Chromatogams of Dendrobium alkaloids. Differences between the accurately tested and calculated m/z values were <2 ppm for [M+H]+ions: (1) Dendrobine, (2) Dendrobine-N-oxide, (3) Nobilonine, (4) Dendroxine, (5) 6-Hydroxy-nobilonine, and (6) 13-Hydroxy-14-oxodendrobine.

Dendrobium was collected from Dendrobium planting regions of Xintian Traditional Chinese Medicine Industry Development co., LTD of Guizhou Province in 2014. The dried stems of the herb (10 kg) were extracted by 95% ethanol solution. DNLA was isolated from the extracts, and analyzed by LC-MS/MS. Alkaloids accounted for 79.8% of DNLA, and mainly contained Dendrobine (C_16_H_25_O_2_N, 92.6%), Dendrobine-N-oxide (C_16_H_25_O_3_N, 3.3%), Nobilonine (C_17_H_27_O_3_N, 2.0%), Dendroxine (C_17_H_25_O_3_N, 0.9%), 6-Hydroxy-nobilonine (C_17_H_27_O_4_N, 0.32%), and 13-Hydroxy-14-oxodendrobine (C_16_H_23_O_4_N, 0.07%). The chemical structures of these ingredients were shown in the Fig. 1A, and the chromatograms of the sample solutions were shown in the Fig. 1B.

Aβ_25–35_ (Sigma, St Louis, MO, USA) was dissolved in physiological saline to a final concentration of 2.5 g/L, and incubated for one week at 37 °C to reach a state of aggregation. CNTF antibodies (ab190985) and GDNF antibodies (ab18956) were obtained from Abcam (Cambridge, England). BDNF (sc-546) was purchased from Santa Cruz Biotechnologies (Santa Cruz, CA, USA). TUNEL kits (*In Situ* Cell Death Detection Kit and POD) were purchased from Roche (Switzerland).

### Animals

Male Kunming mice (20–25 g) were purchased from the Laboratory Animal Center, Chongqing, China (Grade: specific pathogen-free [SPF], Certificate no.: SCXK 2012-0005). Mice were housed in SPF-grade animal facilities (Certificate no.: SYXK 2014-003), with 22–23 °C, and a 12-hour light/dark cycle. Mice were given food and water freely. All animal procedures were approved by the animal experimental ethical committee of Zunyi Medical College.

### Experimental designs

The animals were randomly divided into three groups (*n* = 6): sham surgery, model, and DNLA (40 mg/kg) treatment group. Mice were anesthetized with 7% chloral hydrate (35–45 mg/kg, *i*.*p*.) and fixed on a stereotaxic instrument (RWD Life Science, Guangdong, China). Then Aβ_25–35_ (2.5 µg/µL, 2 µL) was injected into both lateral cerebral ventricle in the model and DNLA groups *via* a 5-µl microinjector. The injection site was posterior from the bregma (AP) = −0.4, mediolateral from the midline (MR) = 1.2, and dorsoventral from the skull (DV) = 2.7. Mice in the sham group were treated the same as the model group except for the injection of Aβ_25–35_ instead of sterile normal saline (Nie et al., 2010). Mice in the DNLA group were administered intragastrically with DNLA (40 mg/kg) on the first day after the injection for 19 consecutive days, while mice in the sham and model groups were administered with distilled water.

### Morris water maze test

The ability of spatial learning and memory of the mice was evaluated through the Morris Water Maze (MWM) test (Edwards et al., 2014; Milner et al., 2014). The test was performed on the 14th day after drug administration. The pool of MWM was 120 cm in diameter, filled with water (22–25 °C) and divided into four quadrants. A hidden platform was placed in the center of the target quadrant 1.5–2.0 cm under the surface of the water. MWM included the place navigation test and the spatial probe test. In the place navigation test, all mice were trained three times per day for four days. Mice were initially placed in the three quadrants which did not have a platform. Each trail lasted for 60 s or ended as soon as the mouse climbed on the platform. The time was recorded as the escape latency, if the mouse climbed on the platform within 60 s. If the mouse failed to find the platform within 60 s, its escape latency was recorded as 60 s. The spatial probe test was performed following the end of the place navigation test. In this test, the platform was removed, and each mouse was allowed to swim for 60 s. The searching distance in the target area and total area were measured. Data was recorded and analyzed by Top View Animal Behavior Analyzing System (Version 3.00).

### Morphometric analysis

After the MWM test, three mice were selected randomly from each group and anesthetized with 7% chloral hydrate. Then these mice were perfused with phosphate-buffered saline (0.1 M, 4 °C) *via* the ascending aorta, followed by 4% paraformaldehyde until the tail and limbs were rigid. Thereafter, the brains were removed and cut in half. Half of the brain was fixed with 4% paraformaldehyde for seven days and cut into coronal sections (4-µm thick) for hematoxylin-eosin (H&E), Nissl and TUNEL staining (Yang et al., 2014; Liu et al., 2015). The other half was fixed with 2.5% glutaraldehyde for electron microscope detection.

### Western blot analysis

Western blot analysis was performed as previously reported (Liu et al., 2015; Li et al., 2015). Three mice from each group were sacrificed after WMW test, and the hippocampal and cortical tissues were collected and homogenized in RIPA lysis buffer (1:5, w/v). Protein concentrations were determined by BCA protein assay kit. An aliquot of 45 µg of protein was applied for electrophoresis followed by the transferring of protein to the polyvinylidene difluoride (PVDF) membranes. The membranes were blocked with 5% nonfat dry milk in TBST buffer for one hour at room temperature, and incubated with the primary antibody: anti-CNTF (1:1,000), BDNF (1:1,000), and GDNF (1:1,000) at 4 °C overnight. Next, HRP-labeled goat anti-rabbit IgG (Beyotime Biotechnology, Jiangsu, China; A0208, 1:2,000) was incubated with the membrane at room temperature for one hour. The blots were visualized using the enhanced ECL Western blot detection kit (7Sea Biotech, Shanghai, China) and scanned to Gel Imaging. The band intensity was quantified using Quantity One 1-D analysis software v4.52 (BioRad, Hercules, California, USA).

### Statistical analysis

All data were presented as mean ± standard error. Data were analyzed by SPSS 13.0 statistics software by one-way ANOVA or student’s *t*-test. *P* < 0.05 was considered as statistically significant.

## Results

### Protective effects of DNLA on learning and memory deficits induced by Aβ_25–35_

**Figure 2:**
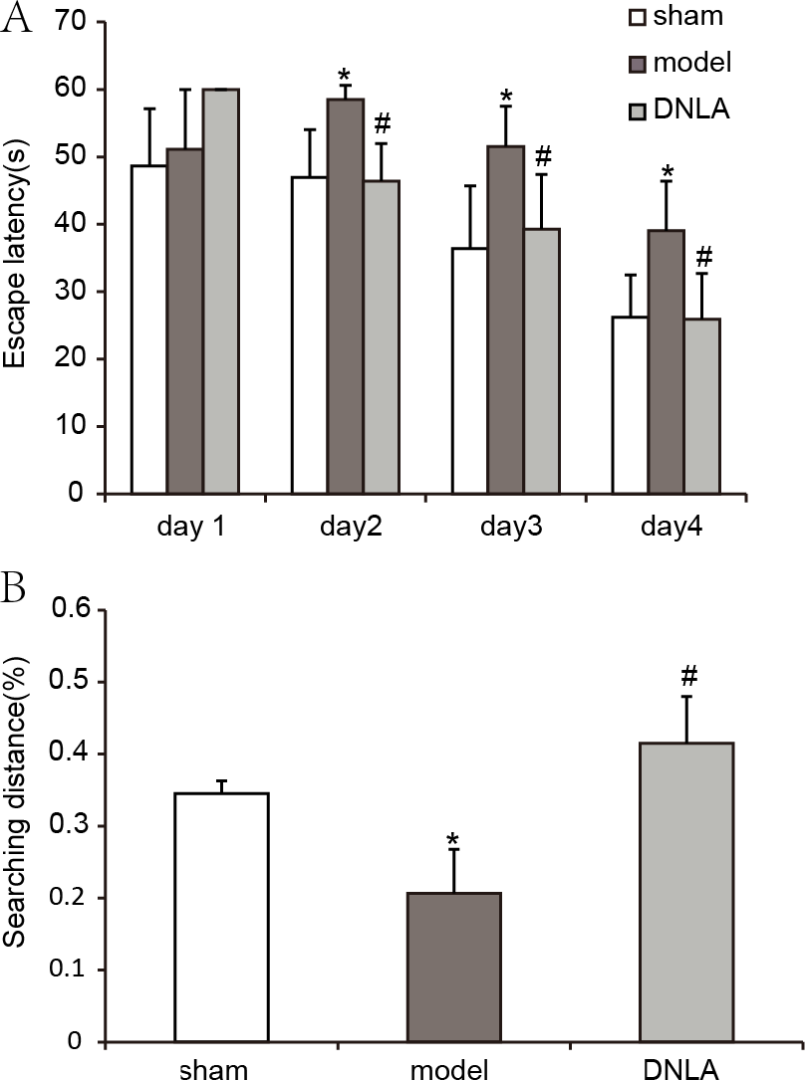
Results of the Morris water maze test. Effect of DNLA on A*β*_25−35_-induced learning and memory impairment. (A) The graph represented the escape latency of the different groups. (B) Graphs described the adjusted searching distance in the space probe test. Data were expressed as mean ± SEM (*n* = 6). ^∗^*P* < 0.05 * vs.* the sham group; ^#^*P* < 0.05 * vs.* model.

Compared with the sham group, the escape latency of the model group was significantly prolonged in the navigation test, and the explored distance in the target area was decreased in the spatial probe test. After treatment for 14 days, the escape latency of the DNLA group was decreased and the explored distance in the target area was increased as compared with the model group. The results indicated that DNLA could protect the mice against learning and memory deficits which were induced by Aβ_25–35_ injection (Fig. 2).

### Morphological changes of cortex and hippocampus tissues

**Figure 3:**
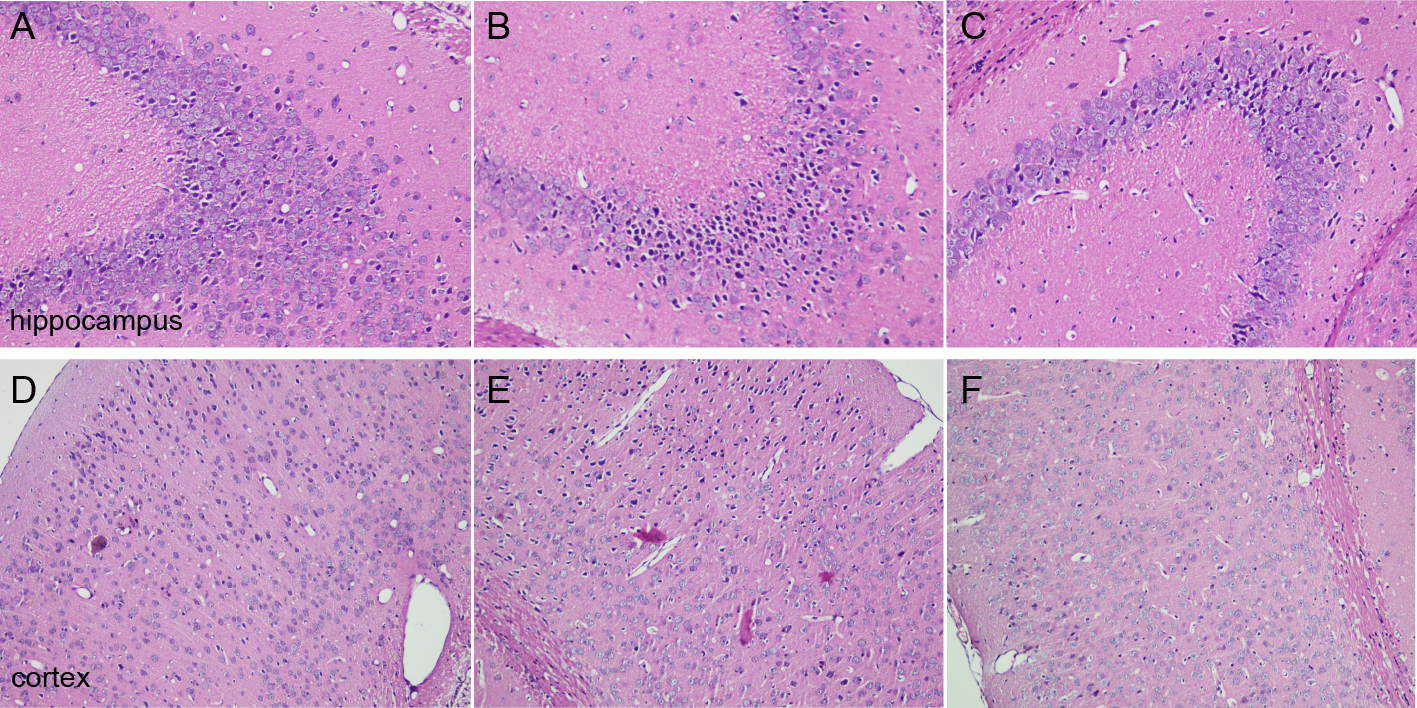
Images of H&E staining. Effects of DNLA on morphological alterations in the hippocampus and cortex induced by A*β*_25−35_. Sections of the hippocampus CA3 region and cortex were obtained and stained with H&E (magnification, 200×). (A–C) showed the CA3 hippocampal cyto-architecture of mice in sham, model and DNLA groups, respectively; (D–F) showed the cortical cyto-architecture of mice in the sham group, model group and DNLA groups, respectively.

H&E staining was performed to observe the morphological changes of cortex and hippocampus tissues in mice brain. The results showed that the morphology of brain tissues in the sham group was normal, and the cell density was higher. However, loose structures in hippocampal and cortical neurons with disordered hippocampal pyramidal cell layers occurred in the model group. Furthermore, the images showed that not only the number of neurons was reduced, but also that neurons staining were abnormal with nuclear condensation in the model group. DNLA treatment significantly decreased the number of abnormally stained neurons. Also, the morphology of brain tissues was generally normal in the DNLA group (Fig. 3).

### Effect of DNLA on apoptosis in the cortex and hippocampus

**Figure 4:**
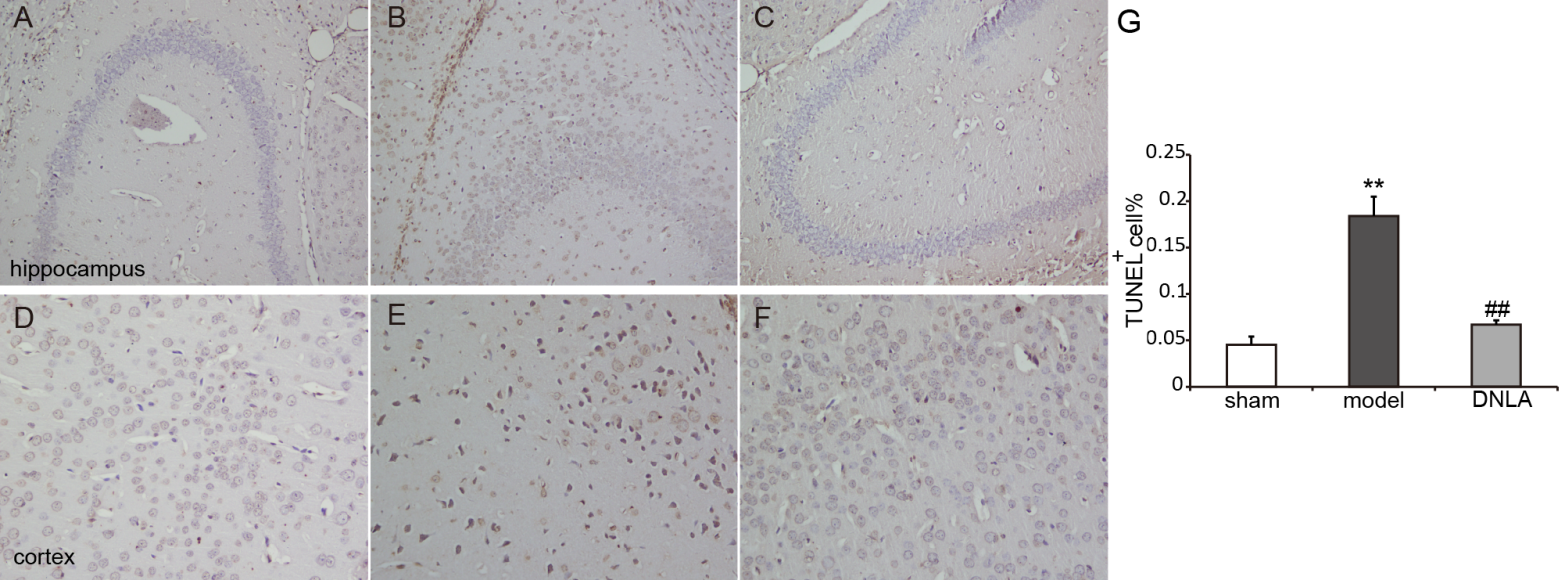
Images of TUNEL staining. Effects of DNLA on A*β*25-35-induced neuronal apoptosis in hippocampus and cortex. Sections of the hippocampus CA3 region and cortex were obtained and stained with TUNEL 184 staining (magnification, 200×). (A–C) showed the CA3 hippocampal cyto-architecture of mice in sham, model and DNLA groups, respectively; (D–F) showed the cortical cyto-architecture of mice in the sham group, model group and DNLA groups, respectively. The cells dyed brown are apoptotic cells. (G) The number of the apoptotic neurons in hippocampus and cortex ($\bar {x}\pm ~SEM,n=3$). **P < 0.01 vs. the sham group; ##*P* < 0.01 vs. the model group.

The effect of DNLA on neuronal apoptosis induced by Aβ_25–35_ was observed using the TUNEL method. The results revealed that the number of apoptotic cells in the hippocampus and cortex were not affected in the sham group, but were increased in the model group. DNLA treatment decreased the number of apoptotic cells (Fig. 4).

### Changes in Nissl bodies in cortex and hippocampus tissues

**Figure 5:**
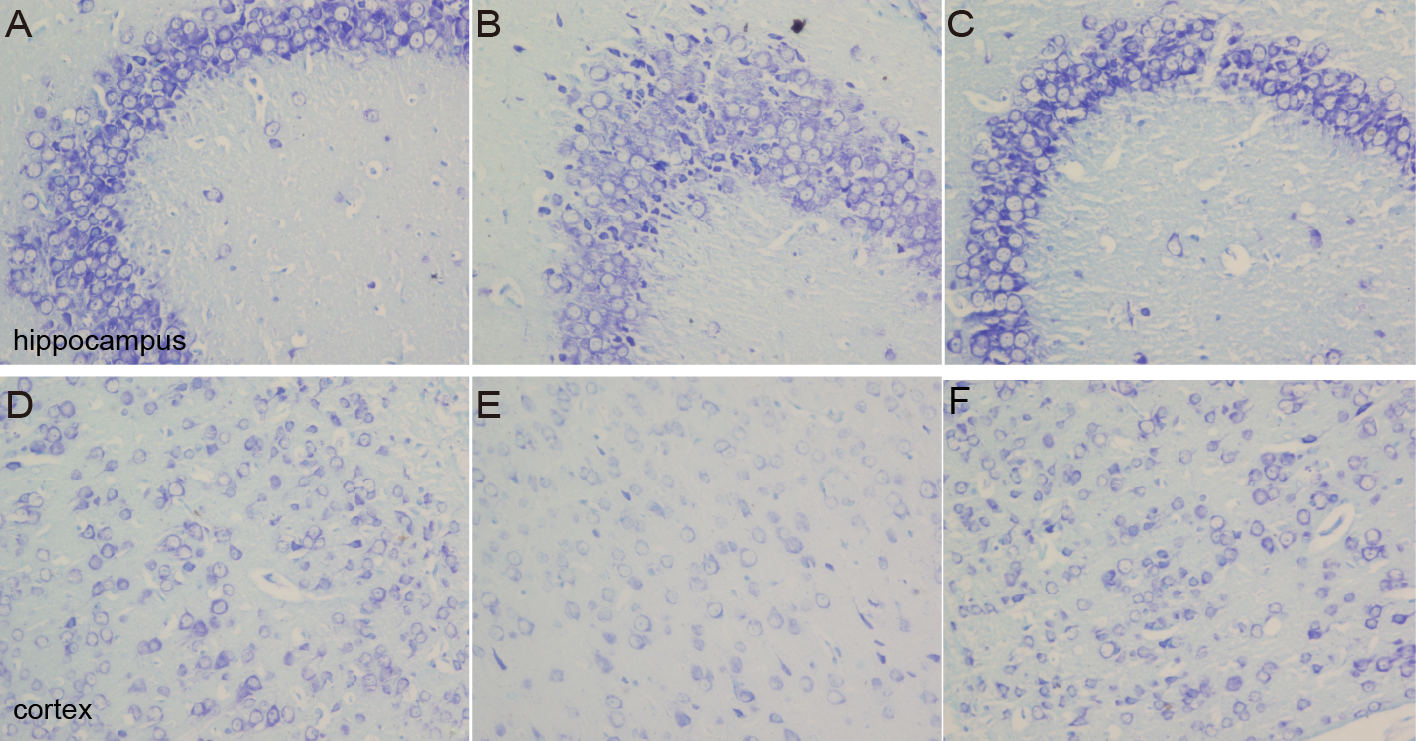
Images of Nissl staining. Effects of DNLA on A*β*_25−35_-induced neuronal Nissl bodies in the hippocampus and cortex. Sections of the hippocampus CA3 region and cortex were obtained and stained with Nissl staining (400×). (A–C) showed the CA3 hippocampal cyto-architecture of mice in sham, model and DNLA groups, respectively; (D–F) showed the cortical cyto-architecture of mice in the sham group, model group and DNLA groups, respectively.

As a neural characteristic structure, the number of Nissl bodies reflects the state of neurons. In physiological conditions, the Nissl bodies were big and abundant, showing that the function of neuronal protein synthesis was strong; on the other hand, when nerve cells were damaged, the number of Nissl bodies will be reduced or even disappear. Intraneural Nissl bodies of the cortex and hippocampus were lightly stained and appeared to be sparsely arranged in the model group. However, deeper stained Nissl bodies with higher density in cortex and hippocampus neurons were found in the sham and DNLA groups (Fig. 5).

### Effects of DNLA on the ultrastructures of neurons in Aβ_25–35_-injected mice

**Figure 6:**
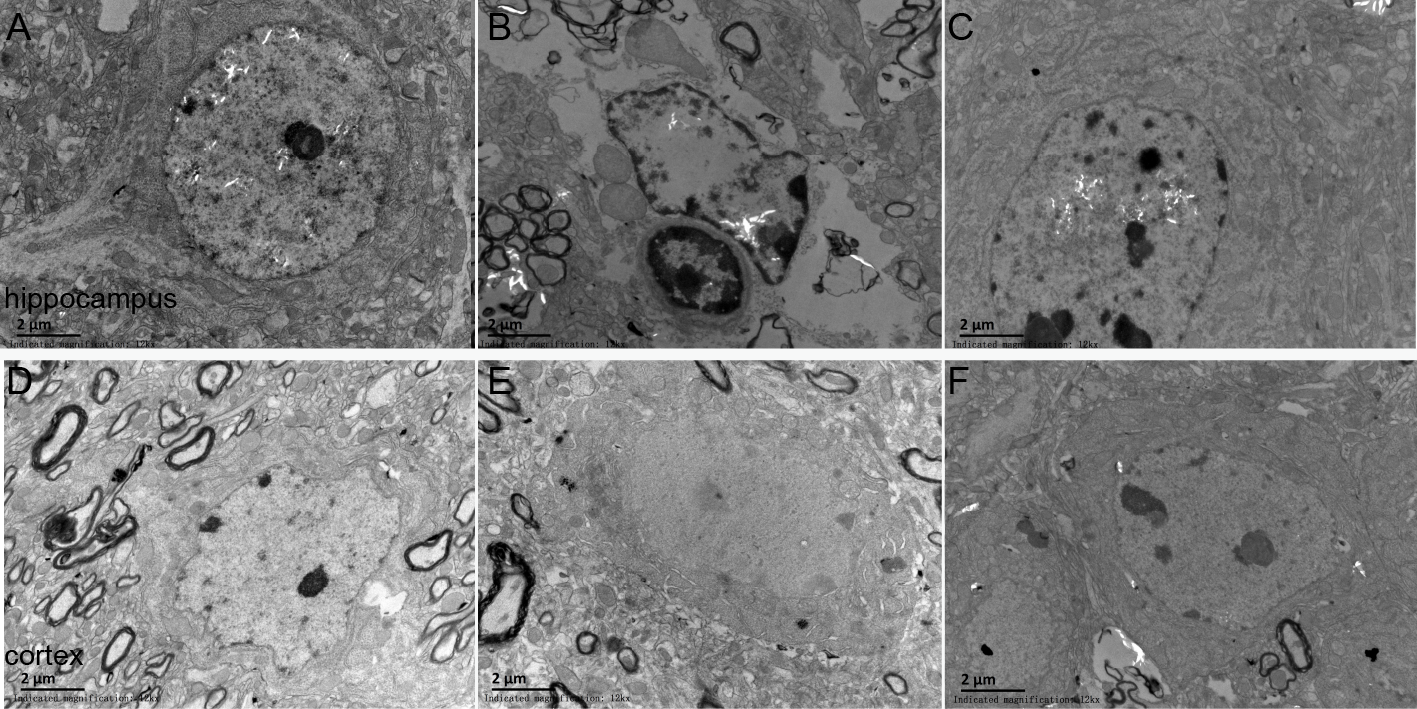
Images of the ultrastructures of neurons. Effects of DNLA on the ultrastructures of neurons in A*β*_25−35_-injected mice. The ultrastructures were observed under electron microscope (12kx ×). (A–C) showed the ultrastructures of hippocampal neurons in sham, model and DNLA groups, respectively; (D–F) showed the ultrastructures of cortical neurons in sham, model and DNLA groups, respectively.

**Figure 7:**
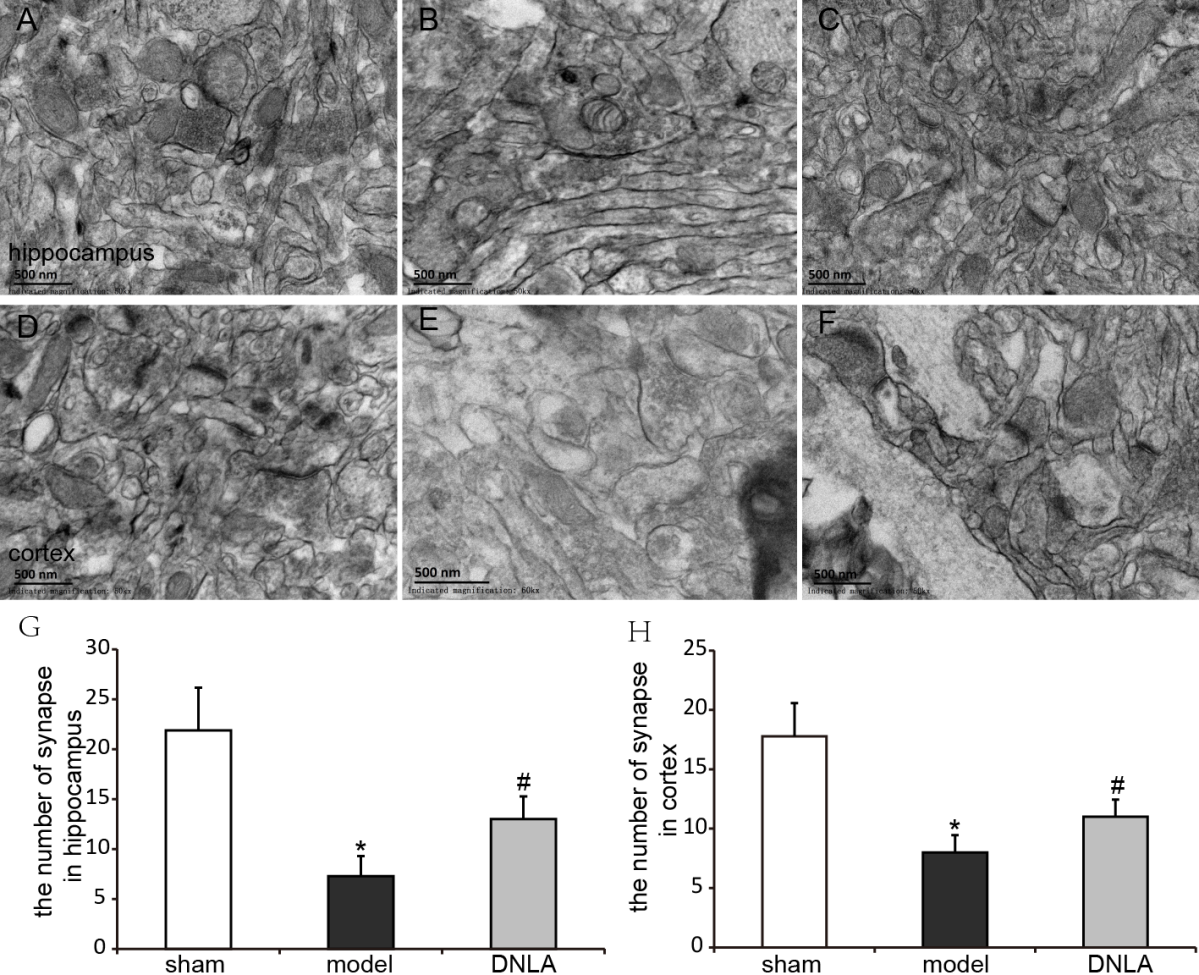
Effects of DNLA on neuronal synapses in Aβ_25–35_-injected mice. The neuronal synapses were observed under electron microscope (50kx ×). (A–C) showed the synapses of hippocampal neurons in sham, model and DNLA groups, respectively; (D–F) showed the synapses of cortical neurons in the sham, model and DNLA groups, respectively. (G) showed the effects of DNLA on the number of neuronal synapses in A*β*_25−35_-induced mice. Data were expressed as mean ± SEM (*n* = 3). ^∗^*P* < 0.05 *vs.* the sham group; ^#^*P* < 0.05 *vs.* the model group.

The ultrastructures of neurons were observed under electron microscope. The results revealed that the nuclear morphology of mice in the sham group was normal, and the chromatin was normally distributed. In the cytoplasm, mitochondrial morphology was normal with clear and regularly-arranged cristae. Furthermore, a number of ribosomes could be observed in these regularly-arranged rough endoplasmic reticulum. However, in the model group, the ultrastructures of neurons were abnormal. It could be found that nuclear membrane had folded or had obscure boundaries, mitochondria were swelling even without the cristae, and the endoplasmic reticulum was swelling. DNLA treatment could reduce the injury. Compared with the model group, nuclear membrane was more smooth, swelling of mitochondrial was reduced, and partial rupture of cristae were observed in the DNLA group (Fig. 6). The neuronal synapses were also observed under electron microscope. Injection of Aβ_25–35_ would reduce the number of synapses and synaptic vesicles, and cause the shape and size of synaptic vesicles uneven. The structure of synapses was obscured, and the structure of the post-synaptic lattice became thin. DNLA inhibited the loss of synapses significantly (*P* < 0.05). Also, in the DNLA group, the generally normal synaptic structures were maintained (Fig. 7).

### Effects of DNLA on the protein expressions of BDNF, CNTF and GDNF in the cortex and hippocampus

**Figure 8:**
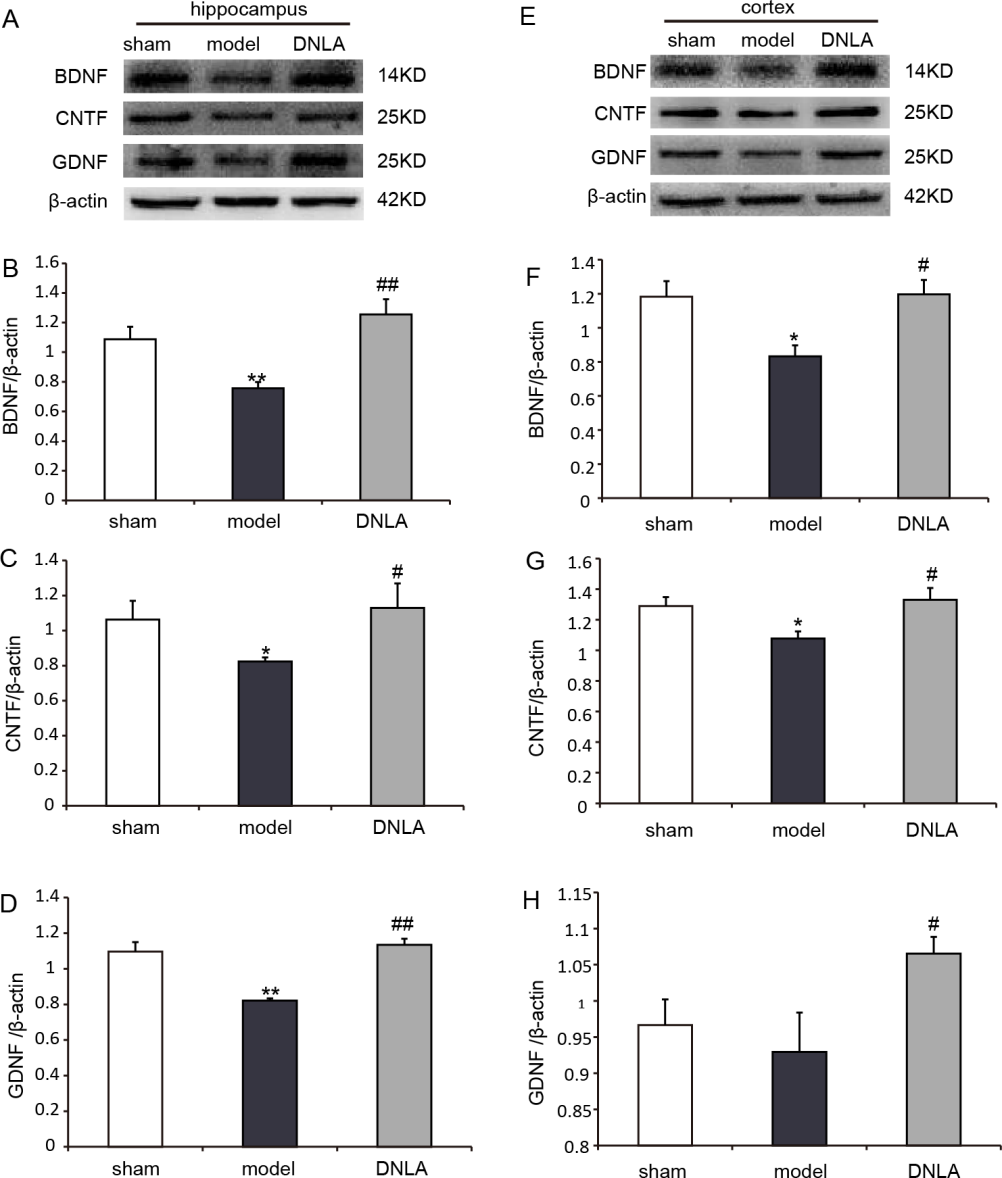
Results of western blot. Effects of DNLA on the protein expressions of BDNF, CNTF and GDNF in brain tissues. (A) Protein contents of hippocampal tissues were plotted for sham, model and DNLA groups, respectively. The corresponding quantitation of BDNF, CNTF, GDNF protein were showed in (B–D) respectively. (E) Protein contents of cortex tissues were plotted for sham, model and DNLA groups, respectively. The corresponding quantitation of BDNF, CNTF, GDNF protein were showed in (F–H) respectively. The relative optical density was normalized to *β*-actin. Data were expressed as mean ± SEM (*n* = 3). Significance ^∗^*P* < 0.05, ^∗∗^*P* < 0.01 compared to the sham group, ^#^*P* < 0.05, ^##^*P* < 0.01 compared to the model group.

The protein expressions were analyzed by Western blot. Compared with the sham group, the expressions of BDNF, CNTF and GDNF significantly decreased in the model group. DNLA group had significantly increased expressions of BDNF, CNTF and GDNF in the cortex and hippocamous (Fig. 8).

## Discussion

Numerous literatures have reported that intra-hippocampal or intra-cerebroventricular injections of Aβ_1–42_ or Aβ_25–35_ fragments into rats or mice could induce neuronal death, and alter spatial learning and memory performance. A*β* could induce a variety of injuries, such as oxidative injury and disturbed neuronal ionic homeostasis, which could eventually result in neuronal dysfunction and selective neuronal loss (Lue et al., 1999; Niikura et al., 2002). Excessive neuronal loss can damage brain functions, and the cognitive ability is destroyed firstly. In this study, we established the mice model which was induced by the intra-cerebroventricular injection of Aβ_25–35_, and found that there were a lot of apoptotic neurons in the hippocampus CA3 region and cortex. The results of MWM test indicated that neuronal apoptosis was accompanied with an obvious failure in spatial learning and memory performance. On the other hand, DNLA treatment could significantly improve the ability of spatial learning and memory, and decreased apoptosis in TUNEL and H&E staining. This result demonstrated that DNLA could improve learning and memory deficits in AD model mice, and the protection may be due to the decreased apoptosis induced by Aβ_25–35_.

As it is known, when neurons are damaged, Nissl bodies are most sensitive. The main changes of Nissl bodies associated with neuronal injury include the dissolution and disappearance of Nissl bodies. In the model group, the number of Nissl bodies was decreased in cells. After the administration of DNLA, the number was increased. The results indicated DNLA could alleviate neuronal damage.

It has reported that memory disorders in AD patients begin with subtle changes in hippocampal synaptic efficacy earlier than extensive neuronal degeneration. Davies et al. (1987) performed a quantitative morphometric analysis to measure the densities of neurons and synapses in cerebral cortical biopsy tissues collected from AD patients. They found that the numerical density of synapses decreased 25%–35%, and the number of synapses per cortical neuron decreased 15%–35% in AD patients (Davies et al., 1987). Evidence also suggested that synaptic dysfunction is caused by oligomeric assemblies of A*β* (Lacor et al., 2004; Rowan et al., 2007). Even in very mildly impaired patients, the degree of synaptic loss in the cortex showed a significant association with soluble A*β* levels. Our results revealed that Aβ_25–35_ induced synapse loss in the hippocampus and cortex, and DLNA could dramatically inhibit the loss of synapses and improve synaptic structures, thereby improving the learning and memory abilities of mice.

The application of neurotrophic factors could enable the modulation of neuronal survival and synaptic connectivity (Poo, 2001). Neurotrophic factors are synthesized and secreted by the target tissue. After binding with receptors (tyrosine receptor kinase [Trk] and pan-neurotrophin receptot [p75]), these factors are internalized and transported to the cell body in a retrograde manner, where they would affect neuronal survival and differentiation (Poo, 2001; Barker & Shooter, 1994). The loss of neurons in the AD process is at least partly due to the lack of one or more neurotrophic factors. It has been found that the mRNA expression level of BDNF is low in the hippocampus and cortex of AD patients. Christensen et al. (2008) reported that the injection of Aβ_1–42_ in the hippocampus of rat would lead to learning and memory impairment and reduce cortical BDNF levels. These results indicate that memory deficit induced by Aβ_1–42_ is associated with the disorder of the expression of BDNF, which is reflected in lower cortical BDNF levels. Another study has shown that secretory vesicles containing BDNF exist within axon terminals, dendrites of pyramidal, and granule cells. BDNF can be secreted into the synaptic gap and binds with TrkB. After binding, BDNF serves as a key regulator in synaptic plasticity and memory, and plays a role in long-term and short-term memory (Lu, Christian & Lu, 2008). The present study proved that intracerebroventricular injection of Aβ_25–35_ could decrease the expression of BDNF in the hippocampus and cortex. However, DNLA have significantly increased BDNF expression, suggesting that the abilities of DNLA to reduce neuronal apoptosis and synaptic loss in mice may be partly associated with increased BDNF protein level.

The maintenance of the neuronal survival and function needs simultaneous support from a variety of neural survival factors, which form an information network through signal transduction pathways in nerve cells, and generate an amplified effect *via* intracellular generalization (Poo, 2001). Therefore, we examined two other neurotrophic factors, CNTF and GDNF. CNTF is a member of a cytokine family (Duff & Baile, 2003), and is known for its neuroprotective effects, being as a survival factor for sympathetic, sensory and hippocampal neurons. Garcia et al. reported that CNTF reduced the impairments of synaptic and cognitive functions in the Tg2576 AD mouse model, and rescues neurons from degeneration induced by A*β* in vitro and in vivo (Pasquin, Sharma & Gauchat, 2015; Garcia et al., 2010). GDNF family and its different receptor systems are now recognized as one of the major neurotrophic networks in the nervous system, which are important for the development, maintenance and function of neurons and glial cells. GDNF signaling through NCAM can result in the activation of kinases MAP and Src-like kinases, and contributes to the regulation of several different processes, including synapse formation and neurite outgrowth (Allen et al., 2013). In hippocampal neuronal cultures, GDNF has been found to increase the number of synapses. Although most studies on the function of GDNF and its receptors has been concentrated on midbrain dopaminergic neurons, the receptors of GDNF are observed in many other brain regions. A study analyzed 250 blood plasma samples of AD patients, and identified GDNF as one of 18 signaling proteins. GDNF level was lower in AD patients than the controls. It is not difficult to envision how a signaling molecule such as GDNF, which has potent effects on neuronal maturation, cell survival and synapse formation, can effect cognitive functions; and if aberrantly or insufficiently expressed or secreted, contribute to cognitive decline (Ibáñez & Andressoo, 2016; Pertusa et al., 2008). Our study also found DNLA can significantly increase the expression of CNTF and GDNF. The results indicated that the ability of DNLA to prevent neuronal and synaptic loss may be attributed to increase the expression of BDNF, CNTF and GDNF.

**Figure 9:**
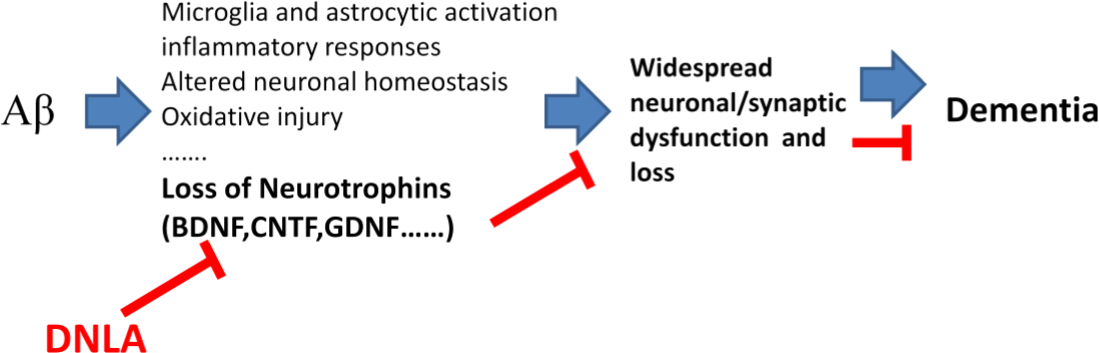
Overview diagram illustrating the neuronal damage induced by A*β*_25−35_ and DNLA intervention. DNLA could prevent neuronal apoptosis and synaptic loss *via* increasing the expression of BDNF, GDNF and CNTF in the hippocampus and cortex, improving A*β*-induced spatial learning and memory impairment in mice.

In summary, the present study demonstrates that DNLA could improve A*β*-induced learning and memory impairment in mice. This may be due to the prevention of neuronal apoptosis and synaptic loss via the increased expression of BDNF, GDNF and CNTF in the hippocampus and cortex (Fig. 9).

##  Supplemental Information

10.7717/peerj.2739/supp-1 Figure S1 Full-length uncropped blots The figures are full-length uncropped blots in western blot.The consequences of sample are sham,model and DNLA group seperately.

10.7717/peerj.2739/supp-2 Data S1 Raw data of the place navigation test in day 1 The file shows the raw data of the place navigation test in day 1. Every mouse have trailed three times, each trail lasted for 60 s or ended as soon as the mouse climbed on the platform. The time was recorded as the escape latency, if the mouse climbed on the platform within 60 s. If the mouse failed to find the platform within 60 s, its escape latency was recorded as 60 s.

10.7717/peerj.2739/supp-3 Data S2 Raw data of the place navigation test in day 2 The file shows the raw data of the place navigation test in day 2. Every mouse have trailed three times, each trail lasted for 60 s or ended as soon as the mouse climbed on the platform. The time was recorded as the escape latency, if the mouse climbed on the platform within 60 s. If the mouse failed to find the platform within 60 s, its escape latency was recorded as 60 s.

10.7717/peerj.2739/supp-4 Data S3 Raw data of the place navigation test in day 3 The file shows the raw data of the place navigation test in day 3. Every mouse have trailed three times, each trail lasted for 60 s or ended as soon as the mouse climbed on the platform. The time was recorded as the escape latency, if the mouse climbed on the platform within 60 s. If the mouse failed to find the platform within 60 s, its escape latency was recorded as 60 s.

10.7717/peerj.2739/supp-5 Data S4 Raw data of the place navigation test inday 4 The file shows the raw data of the place navigation test in day 4. Every mouse have trailed three times, each trail lasted for 60 s or ended as soon as the mouse climbed on the platform. The time was recorded as the escape latency, if the mouse climbed on the platform within 60 s. If the mouse failed to find the platform within 60 s, its escape latency was recorded as 60 s.

10.7717/peerj.2739/supp-6 Data S5 Raw data of the spatial probe test The file shows the raw data of the spatical probe test. In this test, the platform was removed, and each mouse was allowed to swim for 60 s, the searching distance in the target area and total area were measured. The target area is ’center in 3’ of the file.

10.7717/peerj.2739/supp-7 Data S6 Raw data of TUNEL staining The file shows the raw data of TUNEL staining. 3 animals are picked up randomly from each group and 3 pieces of each brain are required to be stained with TUNEL staining. we have taken photos of them from 3-4 perspectives randomly magnified 400 times.Then, calculated the numbers of total cells and apoptotic cells.

10.7717/peerj.2739/supp-8 Data S7 Raw data of the number of synapse The file shows the raw data of the number of synapse.We observed 3 animals of each groups under the electron microscope and take photos of each from 2-3 perspectives magnified 50kx times. Then, calculate the number of synapse.

10.7717/peerj.2739/supp-9 Data S8 Raw data of western blot The file shows the raw data of band intensity of BDNF,CNTF and GDNF protein expression in cortex.

10.7717/peerj.2739/supp-10 Data S9 Raw data of western blot The file shows the raw data of band intensity of BDNF,CNTF and GDNF protein expression in hippocampus.

## Additional Information and Declarations

### Field Study Permissions

The following information was supplied relating to field study approvals (i.e., approving body and any reference numbers): Dendrobium was collected from Dendrobium planting regions of Xintian Traditional Chinese Medicine Industry Development Co., Ltd of Guizhou Province.

### Data Availability

The following information was supplied regarding data availability: The raw data has been supplied as a Supplementary File.

## References

[ref-1] Allen SJ, Watson JJ, Shoemark DK, Barua NU, Patel NK (2013). GDNF, NGF and BDNF as therapeutic options for neurodegeneration. Pharmacology & Therapeutics.

[ref-2] Barker PA, Shooter EM (1994). Disruption of NGF binding to the low affinity neurotrophin receptor p75LNTR reduces NGF binding to TrkA on PC12 cells. Neuron.

[ref-3] Chen JW, Ma H, Huang XN, Gong QH, Qin W, Shi JS (2008). Improvement of *Dendrobium nobile* Lindl. alkaloids on cognitive deficit in rats induced by lipopolysaccharides. Chinese Journal of Pharmacology and Toxicology.

[ref-4] Christensen R, Marcussen AB, Wörtwein G, Knudsen GM, Aznar S (2008). Abeta(1–42) injection causes memory impairment, lowered cortical and serum BDNF levels, and decreased hippocampal 5-HT(2A) levels. Experimental Neurology.

[ref-5] Davies CA, Mann DM, Sumpter PQ, Yates PO (1987). A quantitative morphometric analysis of the neuronal and synaptic content of the frontal and temporal cortex in patients with Alzheimer’s disease. Journal of the Neurological Sciences.

[ref-6] Duff E, Baile CA (2003). Ciliary neurotrophic factor: a role in obesity?. Nutrition Reviews.

[ref-7] Edwards SR, Hamlin AS, Marks N, Coulson EJ, Smith MT (2014). Comparative studies using the Morris water maze to assess spatial memory deficits in two transgenic mouse models of Alzheimer’s disease. Clinical and Experimental Pharmacology and Physiology.

[ref-8] Garcia P, Youssef I, Utvik JK, Florent-Béchard S, Barthélémy V, Malaplate-Armand C, Kriem B, Stenger C, Koziel V, Olivier JL, Escanye MC, Hanse M, Allouche A, Desbène C, Yen FT, Bjerkvig R, Oster T, Niclou SP, Pillot T (2010). Ciliary neurotrophic factor cell-based delivery prevents synaptic impairment and improves memory in mouse models of Alzheimer’s disease. Journal of Neuroscience.

[ref-9] Haass C, Selkoe DJ (2007). Soluble protein oligomers in neurodegeneration: lessons from the Alzheimer’s amyloid beta-peptide. Nature Reviews Molecular Cell Biology.

[ref-10] Ibáñez CF, Andressoo JO (2016). Biology of GDNF and its receptors—relevance for disorders of the central nervous system. Neurobiology of Disease.

[ref-11] Klafki H-W, Kornhuber J, Staufenbiel M, Wiltfang J (2006). Therapeutic approaches to Alzheimer’s disease. Brain.

[ref-12] Lacor PN, Buniel MC, Chang L, Fernandez SJ, Gong Y, Viola KL, Lambert MP, Velasco PT, Bigio EH, Finch CE, Krafft GA, Klein WL (2004). Synaptic targeting by Alzheimer’s-related amyloid *β* oligomers. Journal of Neuroscience.

[ref-13] Li WX, Deng YY, Li F, Liu B, Liu HY, Shi JS, Gong QH (2015). Icariin, a major constituent of flavonoids from Epimedium brevicornum, protects against cognitive deficits induced by chronic brain hypoperfusion via its anti-amyloidogenic effect in rats. Pharmacology Biochemistry and Behavior.

[ref-14] Li Y, Li F, Gong Q, Wu Q, Shi J (2011). Inhibitory effects of dendrobium alkaloids on memory impairment induced by lipopolysaccharide in rats. Planta Medica.

[ref-15] Liu H, Deng Y, Gao J, Liu Y, Li W, Shi J, Gong Q (2015). Sodium Hydrosulfide attenuates beta-amyloid-induced cognitive deficits and neuroinflammation via modulation of MAPK/NF-*κ*B pathway in rats. Current Alzheimer Research.

[ref-16] Lu Y, Christian K, Lu B (2008). BDNF: a key regulator for protein synthesis-dependen LTP and long-term memory?. Neurobiology of Learning and Memory.

[ref-17] Lue LF, Kuo YM, Roher AE, Brachova L, Shen Y, Sue L, Beach T, Kurth JH, Rydel RE, Rogers J (1999). Soluble amyloid *β* peptide concentration as a predictor of synaptic change in Alzheimer’s disease. The American Journal of Pathology.

[ref-18] Milner E, Holtzman JC, Friess S, Hartman RE, Brody DL, Han BH, Zipfel GJ (2014). Endovascular perforation subarachnoid hemorrhage fails to cause Morris water maze deficits in the mouse. Journal of Cerebral Blood Flow and Metabolism.

[ref-19] Nie J, Luo Y, Huang XN, Gong QH, Wu Q, Shi JS (2010). Icariin inhibits beta-amyloid peptide segment 25–35 induced expression of *β*-secretase in rat hippocampus. European Journal of Pharmacology.

[ref-20] Niikura T, Hashimoto Y, Tajima H, Nishimoto I (2002). Death and survival of neuronal cells exposed to Alzheimer’s insults. Journal of Neuroscience Research.

[ref-21] Pasquin S, Sharma M, Gauchat JF (2015). Ciliary neurotrophic factor (CNTF) New facets of an old molecule for treating neurodegenerative and metabolic syndrome pathologies. Cytokine & Growth Factor Reviews.

[ref-22] Pertusa M, García-Matas S, Mammeri H, Adell A, Rodrigo T, Mallet J, Cristòfol R, Sarkis C, Sanfeliu C (2008). Expression of GDNF transgene in astrocytes improves cognitive deficits in aged rats. Neurobiology of Aging.

[ref-23] Poo MM (2001). Neurotrophins as synaptic modulators. Nature Reviews Neuroscience.

[ref-24] Roberson ED, Mucke L (2006). 100 years and counting prospects for defeating alzheimers disease. Science.

[ref-25] Rowan MJ, Klyubin I, Wang Q, Hu NW, Anwyl R (2007). Synaptic memory mechanisms: alzheimer’s disease amyloid *β*-peptide-induced dysfunction. Biochemical Society Transactions.

[ref-26] Scheff SW, Price DA, Schmitt FA, DeKosky ST, Mufson EJ (2007). Synaptic alterations in CA1 in mild Alzheimer disease and mild cognitive impairment. Neurology.

[ref-27] Selkoe DJ (2002). Alzheimer’s disease is a synaptic failure. Science.

[ref-28] Wang Q, Gong Q, Wu Q, Shi J (2010). Neuroprotective effects of Dendrobium alkaloids on rat cortical neurons injured by oxygen-glucose deprivation and reperfusion. Phytomedicine.

[ref-29] Yang S, Gong Q, Wu Q, Li F, Lu Y, Shi J (2014). Alkaloids enriched extract from *Dendrobium nobile* Lindl. attenuatestau protein hyperphosphorylation and apoptosis induced bylipopolysaccharide in rat brain. Phytomedicine.

[ref-30] Youssef I, Florent-Béchard S, Malaplate-Armand C, Koziel V, Bihain B, Olivier JL, Leininger-Muller B, Kriem B, Oster T, Pillot T (2008). N-truncated amyloid oligomers induce learning impairment and neuronal apoptosis. Neurobiology of Aging.

[ref-31] Zhang JQ, Wu Q, Gong QH, Zhang F, Jin F, Nie J, Shi JS (2011). Effects of Dendrobium nobile total alkaloids on lipopolysaccharide-induced astrocyte activation and pro-inflammatory factors production. Chinese Pharmacological Bulletin.

