# Supplementary figures and images for: Dendrobium alkaloids prevent Aβ25–35-induced neuronal and synaptic loss via promoting neurotrophic factors expression in mice
Source: PeerJ. 2016 Dec 13;4:e2739. doi: 10.7717/peerj.2739 (PMC5157189; doi:10.7717/peerj.2739)

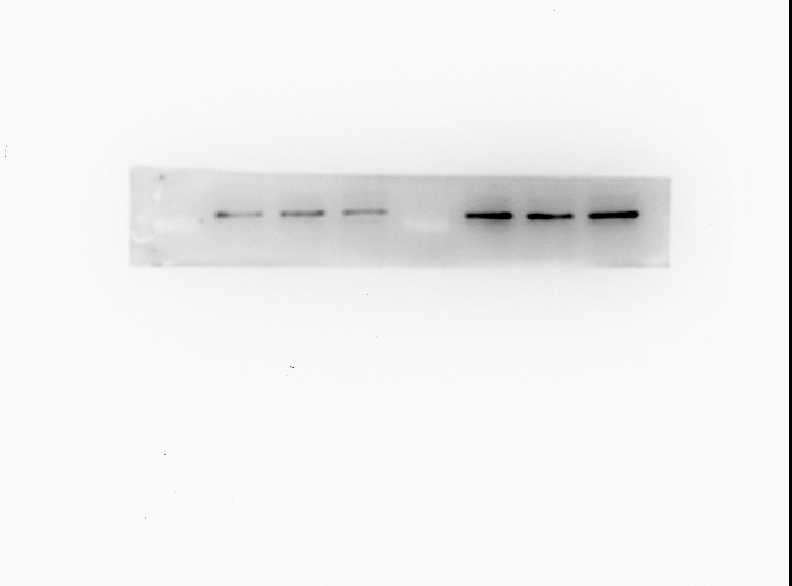

Supplement: Figure S1 — The figures are full-length uncropped blots in western blot.The consequences of sample are sham,model and DNLA group seperately. [file peerj-04-2739-s001.zip › WB figure/C--CNTF.jpg]

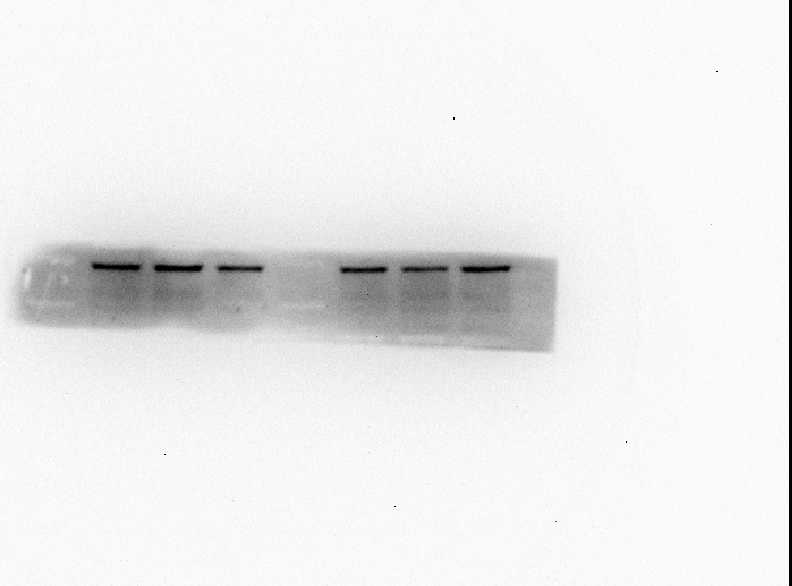

Supplement: Figure S1 — The figures are full-length uncropped blots in western blot.The consequences of sample are sham,model and DNLA group seperately. [file peerj-04-2739-s001.zip › WB figure/C--GDNF.jpg]

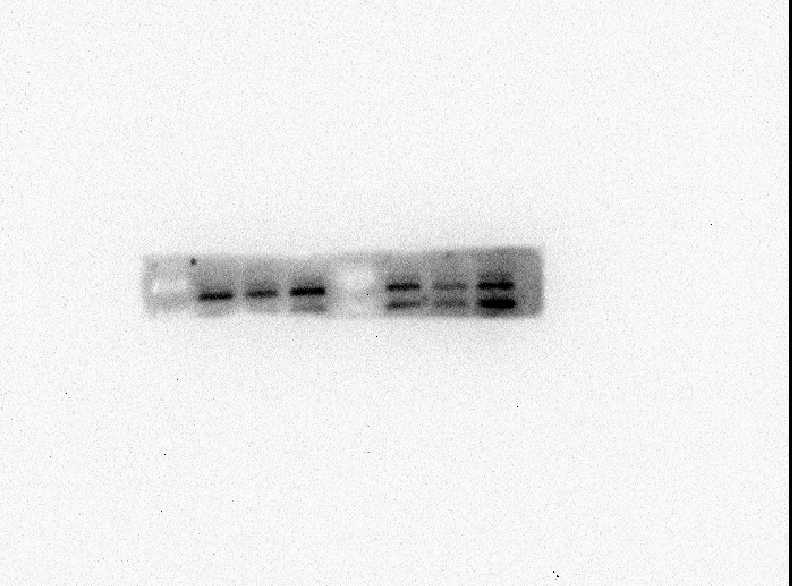

Supplement: Figure S1 — The figures are full-length uncropped blots in western blot.The consequences of sample are sham,model and DNLA group seperately. [file peerj-04-2739-s001.zip › WB figure/C-BDNF.jpg]

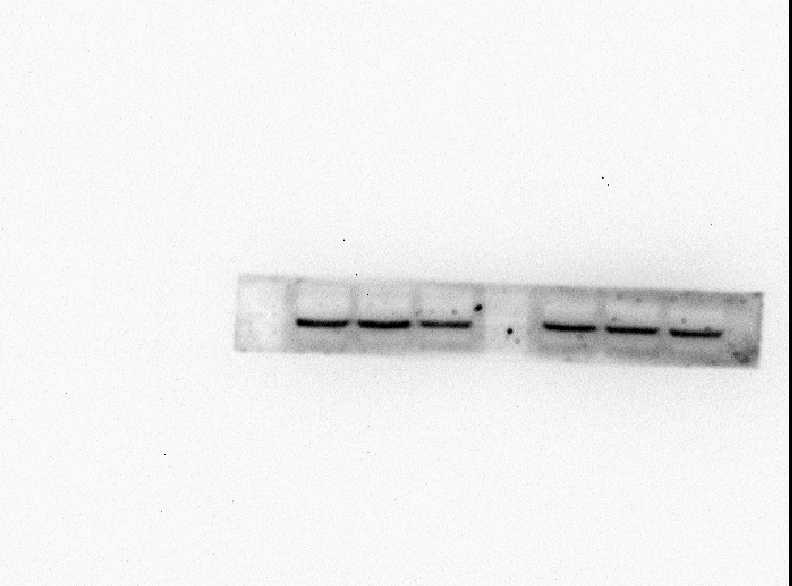

Supplement: Figure S1 — The figures are full-length uncropped blots in western blot.The consequences of sample are sham,model and DNLA group seperately. [file peerj-04-2739-s001.zip › WB figure/C-actin.jpg]

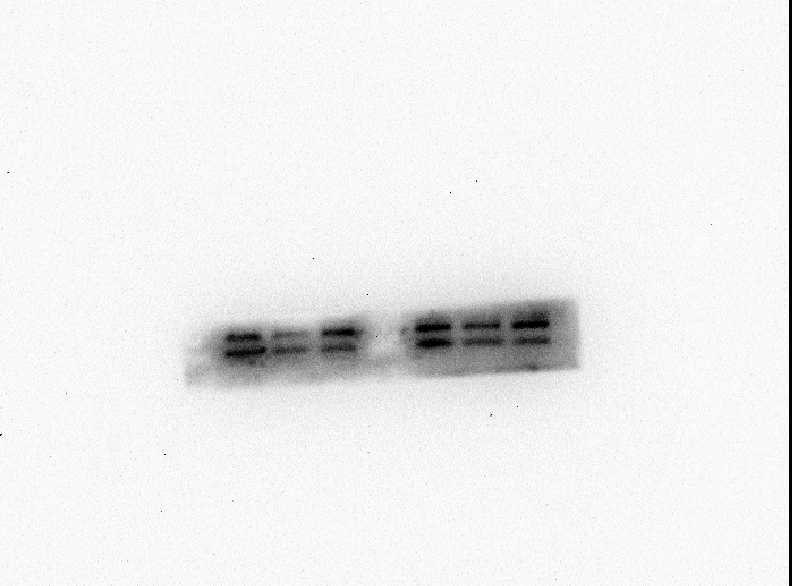

Supplement: Figure S1 — The figures are full-length uncropped blots in western blot.The consequences of sample are sham,model and DNLA group seperately. [file peerj-04-2739-s001.zip › WB figure/H-BDNF.jpg]

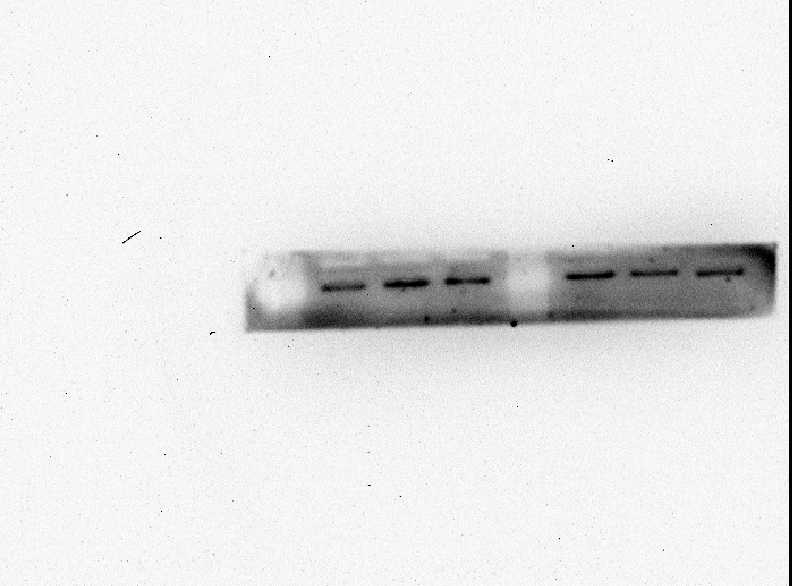

Supplement: Figure S1 — The figures are full-length uncropped blots in western blot.The consequences of sample are sham,model and DNLA group seperately. [file peerj-04-2739-s001.zip › WB figure/H-CNTF.jpg]

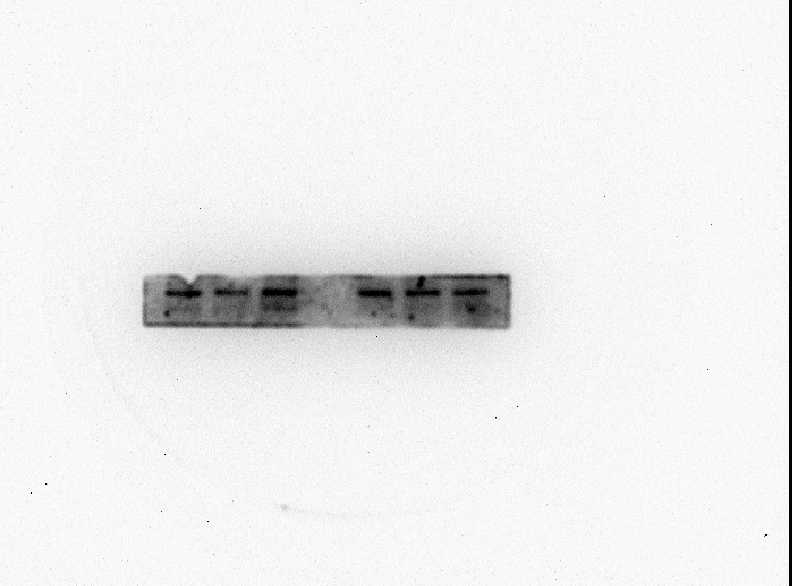

Supplement: Figure S1 — The figures are full-length uncropped blots in western blot.The consequences of sample are sham,model and DNLA group seperately. [file peerj-04-2739-s001.zip › WB figure/H-GDNF.jpg]

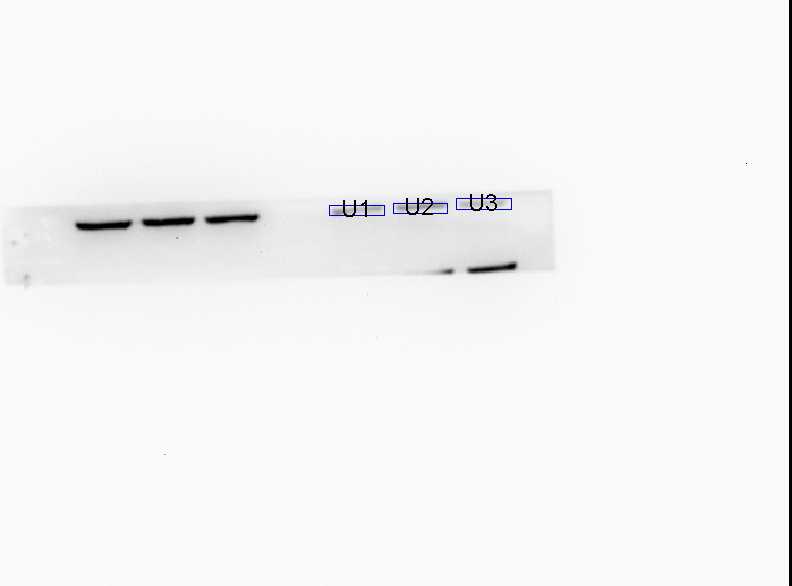

Supplement: Figure S1 — The figures are full-length uncropped blots in western blot.The consequences of sample are sham,model and DNLA group seperately. [file peerj-04-2739-s001.zip › WB figure/H-actin.jpg]
