# Supplementary material for: Dendrobium alkaloids prevent Aβ25–35-induced neuronal and synaptic loss via promoting neurotrophic factors expression in mice
Source: PeerJ. 2016 Dec 13;4:e2739. doi: 10.7717/peerj.2739 (PMC5157189; doi:10.7717/peerj.2739)
Supplement: Data S2 — The file shows the raw data of the place navigation test in day 2. Every mouse have trailed three times, each trail lasted for 60 s or ended as soon as the mouse climbed on the platform. The time was recorded as the escape latency, if the mouse climbed on the platform within 60 s. If the mouse failed to find the platform within 60 s, its escape latency was recorded as 60 s. [file peerj-04-2739-s003.pdf]

**group**	Trial Duration(Second)	Trial Duration(Second)	Trial Duration(Second)
K1	18.40	15.32	60
K2	52.72	60	37.96
K3	60.00	60	45.92
K4	60.00	60	60
K5	19.20	32.68	60
K6	60.00	28.8	60
K7	44.40	16.88	60
M1	23.52	60	60
M2	10.60	59.8	60
M3	60.00	60	60
M4	60.00	60	60
M5	18.32	42.28	60
M6	29.96	60	60
J1	49.32	37.68	52.6
J2	21.28	44.36	60
J3	60.00	31.48	60
J4	60.00	43.76	60
J5	43.20	54.96	31.56
J6	47.60	60	20.52
