# Supplementary material for: Dendrobium alkaloids prevent Aβ25–35-induced neuronal and synaptic loss via promoting neurotrophic factors expression in mice
Source: PeerJ. 2016 Dec 13;4:e2739. doi: 10.7717/peerj.2739 (PMC5157189; doi:10.7717/peerj.2739)
Supplement: Data S5 — The file shows the raw data of the spatical probe test. In this test, the platform was removed, and each mouse was allowed to swim for 60 s, the searching distance in the target area and total area were measured. The target area is ’center in 3’ of the file. [file peerj-04-2739-s006.pdf]

distance(mm)

group	center in basin	center in platform	center in 1	center in 2	center in 3	center in 4	total distance	center in 3/total distance
k1	0	107.81	296.85	258.79	941.18	1165.15	2769.78	0.339803161
K2	0	51.4	483.59	598.53	1517.56	1045.45	3696.53	0.410536368
K3	0	55.66	548.25	809.68	1132.19	481.81	3027.59	0.373957504
K5	0	26.83	763.24	1108.12	1088.36	239.74	3226.29	0.337341033
K5	0	56.11	558.16	1630.77	1045.04	586.59	3876.67	0.269571565
K6	0	0	751.65	1061.59	1398.25	767.5	3978.99	0.351408272
K7	0	20.39	583.64	1190.85	1106.76	401.45	3303.09	0.335068073
M1	0	64.51	273.1	761.86	973.29	336.72	2409.48	0.403941929
M2	0	23.57	775.66	1235.97	494.29	555.76	3085.25	0.16021068
M3	0	0	455.78	647.68	890.28	533.67	2527.41	0.352249932
M4	0	16.41	920.46	1071.74	124.79	620.53	2753.93	0.045313425
M5	0	45.77	1151.58	978.79	844.05	765.71	3785.9	0.222945667
M6	0	0	431.56	572.11	90.31	523.17	1617.15	0.05584516
J1	0	17.12	392.97	737.9	1411.94	329.31	2889.24	0.488689067
J2	0	25.58	745.57	268.1	1145.71	919.78	3104.74	0.369019628
J3	0	19.2	569.17	596.63	778.42	410.79	2374.21	0.327864848
J4	0	68.97	879.34	904.67	1210.61	870.11	3933.7	0.307753515
J5	0	0	889.89	1035.26	1103.54	772.99	3801.68	0.29027693
J6	0	111.4	325.74	132.83	2243.79	363.43	3177.19	0.706218388
