# Supplementary material for: Dendrobium alkaloids prevent Aβ25–35-induced neuronal and synaptic loss via promoting neurotrophic factors expression in mice
Source: PeerJ. 2016 Dec 13;4:e2739. doi: 10.7717/peerj.2739 (PMC5157189; doi:10.7717/peerj.2739)
Supplement: Data S6 — The file shows the raw data of TUNEL staining. 3 animals are picked up randomly from each group and 3 pieces of each brain are required to be stained with TUNEL staining. we have taken photos of them from 3-4 perspectives randomly magnified 400 times.Then, calculated the numbers of total cells and apoptotic cells. [file peerj-04-2739-s007.pdf]

sham the number of apoptotic cells

No. 1	10	13	11	13	8	11	18	20	14	
No. 2	8	11	6	8	14	5	5	17	14	
No. 3	20	11	12	8	6	23	38	18	16	

model

No. 1	65	64	32	20	27	49	52	45	34	34
No. 2	67	79	62	66	77	40	34	28	28	49
No. 3	65	69	33	85	73	62	33	49	41	55

DNLA

No. 1	9	7	6	4	12	5	6	3	9	29
No. 2	26	30	20	13	15	3	2	24	21	3
No. 3	8	13	13	13	28	30	25	28	27	14

sham the number of total cells

No. 1	229	238	200	258	295	313	251	286	219	
No. 2	301	357	293	290	314	341	362	211	293	
No. 3	379	392	348	321	343	420	326	297	305	

model

No. 1	463	425	471	228	383	310	366	329	371	360
No. 2	342	399	341	324	326	359	301	295	343	315
No. 3	278	288	302	302	293	332	214	200	237	252

DNLA

No. 1	307	228	220	214	386	219	259	263	224	279
No. 2	276	315	269	210	224	226	169	227	194	178
No. 3	246	303	297	251	296	275	275	253	227	319
