# Supplementary material for: Dendrobium alkaloids prevent Aβ25–35-induced neuronal and synaptic loss via promoting neurotrophic factors expression in mice
Source: PeerJ. 2016 Dec 13;4:e2739. doi: 10.7717/peerj.2739 (PMC5157189; doi:10.7717/peerj.2739)
Supplement: Data S7 — The file shows the raw data of the number of synapse.We observed 3 animals of each groups under the electron microscope and take photos of each from 2-3 perspectives magnified 50kx times. Then, calculate the number of synapse. [file peerj-04-2739-s008.pdf]

the number of synapse

Cortex

sham	model	DNLA
25	14	15
36	3	12
25	7	13
23	7	10
17	9	22
14	5	13
13	6	8
22		13
		11

Hippocampus

sham	model	DNLA
23	5	9
19	7	11
13	13	9
23	9	9
11	7	10
13	7	14
23	8	15
20		
15		
