# Supplementary material for: Dendrobium alkaloids prevent Aβ25–35-induced neuronal and synaptic loss via promoting neurotrophic factors expression in mice
Source: PeerJ. 2016 Dec 13;4:e2739. doi: 10.7717/peerj.2739 (PMC5157189; doi:10.7717/peerj.2739)
Supplement: Data S8 — The file shows the raw data of band intensity of BDNF,CNTF and GDNF protein expression in cortex. [file peerj-04-2739-s009.pdf]

		actin	cntf	cntf/actin
No1	sham	22.69628	31.32694	1.380267388
	model	16.75303	16.52368	0.986309756
	DNLA	15.62082	22.76984	1.457659759
No2	sham	14.5421	17.13752	1.178476012
	model	15.74549	17.48488	1.110468972
	DNLA	14.64228	17.44363	1.191319445
No3	sham	17.99557	23.51608	1.30677066
	model	18.62562	21.14499	1.135263451
	DNLA	19.90686	26.70578	1.341536327

		actin	BDNF	bdnf/actin
No2	sham	14.5421	16.47172	1.13269183
	model	15.74549	14.93242	0.948361953
	DNLA	14.64228	18.44704	1.259848222
No3	sham	15.49423	16.35932	1.055832501
	model	17.15426	14.14664	0.824671978
	DNLA	19.09801	19.64286	1.028529178
		14.5421	19.76639	1.359252487
No1	sham	15.74549	11.40183	0.724132851
	model	14.64228	19.04442	1.300646254
	DNLA			

		actin	gndf	gdnf/actin
No2	sham	31.72	31.59	0.995901639
	model	35.02	33.01	0.942604226
	DNLA	33.26	35.39	1.06404089
No3	sham	29.59	27.12	0.916525853
	model	33.2	33.66	1.013855422
	DNLA	37.22	39.22	1.053734551
No1	sham	35.35	36.73	1.03903819
	model	35.14	27.93	0.794820717
	DNLA	34.51	35.34	1.024051
No3	sham	32.81	30.01	0.914660165
	model	34.09	32.93	0.965972426

DNLA	33.1	37.06	1.119637462

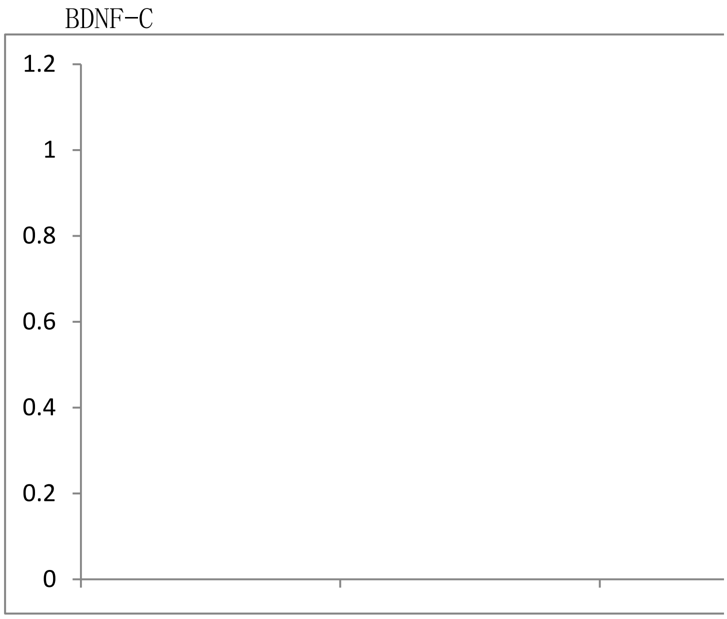
BDNF-C
1.2
1
0.8
0.6
0.4
0.2
0
