# Supplementary material for: Dendrobium alkaloids prevent Aβ25–35-induced neuronal and synaptic loss via promoting neurotrophic factors expression in mice
Source: PeerJ. 2016 Dec 13;4:e2739. doi: 10.7717/peerj.2739 (PMC5157189; doi:10.7717/peerj.2739)
Supplement: Data S9 — The file shows the raw data of band intensity of BDNF,CNTF and GDNF protein expression in hippocampus. [file peerj-04-2739-s010.pdf]

		actin	cntf	cntf/actin
No1	sham	17.86961	17.46731	0.977487
	model	17.20612	14.01675	0.814638
	DNLA	16.29079	16.24006	0.996886
No1	sham	31.09625	41.51058	1.334906
	model	37.39025	32.79485	0.877096
	DNLA	31.5135	27.69457	0.878816
No2	sham	32.23727	32.71278	1.014751
	model	39.2334	32.36904	0.825038
	DNLA	28.52933	34.91818	1.22394
No3	sham	31.56734	29.2191	0.925612
	model	41.20506	32.10653	0.779189
	DNLA	27.2276	38.67436	1.42041

		actin	BDNF	bdnf/actin
No1	sham	31.09625	38.18484	1.227956
	model	37.39025	30.46428	0.814765
	DNLA	31.5135	31.35088	0.99484
No2	sham	32.23727	28.51014	0.884384
	model	39.2334	31.96444	0.814725
	DNLA	28.52933	39.52542	1.385431
No3	sham	31.56734	36.26345	1.148765
	model	41.20506	27.3181	0.662979
	DNLA	27.2276	36.41845	1.337556
No3	sham	31.56734	34.27679	1.085831
	model	41.20506	30.22321	0.733483
	DNLA	27.2276	35.5	1.303824

		actin	gdnf	gdnf/actin
No1	sham	31.09625	32.0773	1.031549
	model	37.39025	30.60937	0.818646
	DNLA	31.5135	37.31333	1.184043

No2	sham	32.23727	34.03815	1.055863
	model	39.2334	33.14214	0.844743
	DNLA	28.52933	32.81971	1.150385
No3	sham	31.56734	37.93668	1.20177
	model	41.20506	32.96595	0.800046
	DNLA	27.2276	29.09737	1.068672

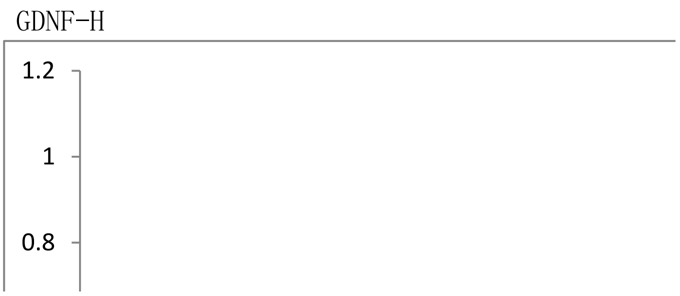
GDNF-H
1.2
1
0.8

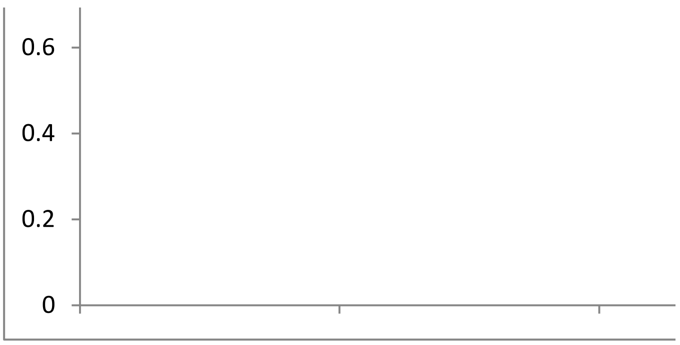
0.6
0.4
0.2
0
